# Congenital thrombotic thrombocytopenic purpura: a rare cause of severe neonatal jaundice and hypoxic respiratory failure – a case report

**DOI:** 10.1515/crpm-2024-0050

**Published:** 2025-07-09

**Authors:** Hilal Al Mandhari, Fatma Albulushi, Nawal Al-Mashaikhi

**Affiliations:** Department of Child Health, 194179 Sultan Qaboos University Hospital, Muscat, Oman; Oman National Centre for Hematology and Marrow Transplantation, Sultan Qaboos University Hospital, Seeb, Oman

**Keywords:** congenital, purpura, thrombotic thrombocytopenic, ADAMTS13 protein

## Abstract

**Objectives:**

This report describes the case of an infant with congenital thrombotic thrombocytopenic purpura.

**Case presentation:**

An infant who presented after birth with severe neonatal indirect hyperbilirubinemia, thrombocytopenia and hemolytic anemia. His initial neonatal course was complicated with hypoxemic respiratory failure due to persistent pulmonary hypertension of the newborn, acute kidney injury and disseminated intravascular coagulopathy. After surviving the acute neonatal presentation, he presented with stress-induced recurrent hemolytic anemia and thrombocytopenia. The diagnosis of congenital TTP was suspected and confirmed by low ADAMTS13 activity, the absence of ADAMTS13 inhibitors, and the identification of a homozygous variant in the *ADAMTS13* gene.

**Conclusions:**

Although rare, congenital TTP needs to be considered by neonatologists when dealing with a neonate with hemolytic jaundice, anemia, and thrombocytopenia. PPHN can complicate the initial presentation of congenital TTP.

## Introduction

Congenital thrombotic thrombocytopenic purpura (c.TTP), or Upshaw-Schulman syndrome, is a rare thrombotic microangiopathy (TMA) entity. It is a life-threatening disease, referring to pathological features of vascular damage. The incidence is estimated at <1 case per million children per year. It is an inherited deficiency of A disintegrin and metalloproteinase with thrombospondin Motifs 13 (ADAMTS13) [1], [2], [3], [4].

In 1960 Schulman et al. described a case of an 8-year-old girl who experienced repeated episodes of thrombocytopenia that improved with plasma infusions. Schulman postulated that a factor in normal plasma that promoted platelet production or maturation was absent in his patient [4]. In 1978, Upshaw described a similar case of a 29-year-old with recurrent episodes of thrombocytopenia associated with microangiopathic hemolytic anemia (MAHA) that also responded to plasma infusions. Upshaw documented 32 episodes of MAHA in his patient over 11 years, the majority of which were preceded by infections or other stressors, such as pregnancy and surgery [5]. In 1982, Moake et al. identified huge von Willebrand Factor (vWF) multimers in the plasma of four patients with chronic relapsing TTP [6]. It has subsequently been demonstrated that ADAMTS13 is the vWF-cleaving protease deficient in c.TTP [7], 8].

The clinical features of c.TTP includes MAHA, consumptive thrombocytopenia, and multi-visceral ischemia. The disease course is characterized by relapsing acute events, mostly triggered by infections and most cases of c.TTP manifests very early after birth. Symptoms typically include jaundice due to severe hemolytic anemia with schizocytosis and a negative Coomb’s test, thrombocytopenia, and some degree of acute kidney injury due to hemoglobinuria. In children with later onset, the first episode is usually triggered by an intercurrent infection. Fever is often present. All have thrombocytopenia with the onset of symptoms and hemolytic anemia coincides or 12–24 h later [9].

This report describes a patient who presented soon after birth with severe indirect hyperbilirubinemia and thrombocytopenia. He had a complicated neonatal course with severe hypoxic respiratory failure secondary to persistent pulmonary hypertension (PPHN), hemolytic anemia, disseminated intravascular coagulopathy (DIC) and acute kidney injury. After surviving the acute neonatal presentation, the diagnosis of c.TTP was established after presenting with recurrent episodes of hemolytic anemia and thrombocytopenia with schizocytosis. The diagnosis was confirmed with low ADAMTS13 activity in the absence of ADAMTS13 inhibitors, and by identifying a homozygous variant c.1192C>G p.(Arg398Gly) in the *ADAMTS 13* gene.

## Case presentation

A term infant was born at 37 weeks of gestation to a 41-year-old, G8P5A2 mother with a history of type II diabetes mellitus (on insulin and metformin), asthma and hypothyroidism (on thyroxine). The family history is positive for a previous child needing phototherapy and negative for any hematological disorders. Her antenatal screening was negative, and her full blood count showed normal platelets. The infant was born via elective cesarean section due to a history of three previous cesarean sections, with Apgar scores of 9 and 10 at 1 and 5 min, respectively. Birth weight was 2.87 kg (∼50th %), head circumference 33 cm (∼50th %), length 51 cm (50–90th %). Postnatally, the infant was active and vigorous and had no respiratory distress. Therefore, he was transferred to the postnatal ward with his mother and started breastfeeding.

The infant was observed to be jaundiced at 8 h of life. The physical examination was normal, except for clinical jaundice. Transcutaneous bilirubin (TCB) was very high at 225 μmol/L; therefore, a full blood count (FBC) and total serum bilirubin (TSB) were performed. The FBC showed normal hemoglobin of 16.2 g/dL, reticulocyte of 6 %, normal WBC and severe thrombocytopenia (platelets 29 × 10^9^/L [RR 150–450 × 10^9^/L]. The TSB was 252 μmol/L, and direct bilirubin was 25 μmol/L (9 % of TSB).

The infant was transferred to the neonatal intensive care unit (NICU) for further care and management. He was immediately started on intensive phototherapy and intravenous fluids. Blood was requested in anticipation of the need for exchange transfusion. Within an hour of admission to the NICU, the baby deteriorated in the form of persistent desaturation with SpO_2_ down to 60 %. Therefore, he was intubated and mechanically ventilated. Post-intubation, he continued to have hypoxemia with pre-ductal SpO_2_ in the 80 % and postnatal SpO_2_ in the 30 % on FiO_2_ of 1.0. Venous blood gas at this stage showed metabolic acidosis with a pH of 7.22, pCO_2_ 25.4 mmHg, HCO_3_ 12 mmol/L, BE -7.7, and lactate 5.3 mmol/L. Chest X-ray showed reduced lung volume, bilateral perihilar shadowing with interstitial markings, mild ground glass appearance, and mild blunting of the right costo-phrenic angle. Considering the clinical picture of hypoxic respiratory failure, urgent echocardiography was performed, which showed persistent pulmonary hypertension with bidirectional shunt across the atrial septal defect and patent ductus arteriosus and moderate left ventricular dysfunction with estimated left ventricular ejection fraction (LVEF) of 25–30 %. After the optimization of mechanical ventilation and administration of surfactant, the infant continued to have hypoxic respiratory failure with worsening metabolic acidosis. Inhaled nitric oxide (iNO) was commenced at 20 ppm. The infant responded positively to iNO; the pre- and post-ductal SpO_2_ difference resolved, pO_2_ increased from 40s to 70 s mmHg, and FiO_2_ gradually weaned down. He had hemodynamic instability, for which he received fluid boluses, inotropic support with dobutamine and adrenaline, as well as hydrocortisone due to catecholamine-resistant shock, after which blood pressure improved. The workup for the indirect hyperbilirubinemia showed no setup for ABO or Rh incompatibility, direct antiglobulin test (DCT) negative and normal G6PD status. The hyperbilirubinemia responded well to intensive phototherapy and exchange transfusion was not required. On day 2 of life, the baby had a clinical picture of intravascular hemolysis (drop in Hb from 16 to 10 g/dL, elevated reticulocyte counts of 7 %, and dark-coloured urine). He received a Packed red blood cells (PRBCs) transfusion. In addition, he had a clinical picture of disseminated intravascular coagulopathy (DIC); thrombocytopenia (lowest 29 × 10^9^/L) and deranged coagulation profile (prolonged PT, INR, APTT, and low fibrinogen). The infant was managed with one platelet transfusion of 10 mL/kg, fresh frozen plasma (FFP) and vitamin K, after which the platelets and coagulopathy improved. Also, he had acute kidney injury (AKI) with a maximum serum creatinine of 130 μmol/L on day four of life. The serum creatinine gradually improved and normalized by day six of life.

Infectious workup showed negative blood culture, enterovirus polymerase chain reaction (PCR), Epstein–Barr virus (EBV) serology, Herpes simplex virus (HSV) PCR, Varicella zoster virus (VZV) PCR, and Parvovirus serology. He received broad-spectrum antibiotics for a total of 10 days. Due to negative workup, the infant was managed as clinical sepsis with secondary pulmonary hypertension and multi-organ dysfunction.

The infant clinically improved with all supportive therapies; iNO was weaned off after 48 h, he was extubated on day 5 of life, inotropes were gradually weaned off, hyperbilirubinemia improved with intensive phototherapy and thrombocytopenia and coagulopathy improved. He was discharged home on the 10th day of life, in a stable clinical condition and on-demand breastfeeding. His full blood count (FBC) before discharge showed Hb 16.1 mg/dL, WBC 11 × 10^9^/L and platelets 133 × 10^9^/L.

**Figure 1:**
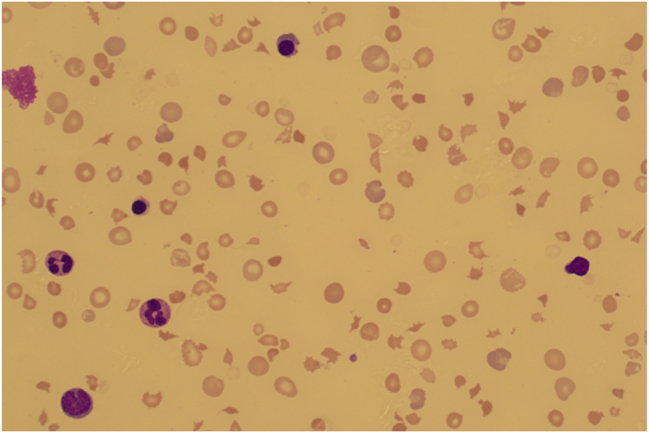
Blood film of the patient showing schistocytes, which are damaged or fragmented red blood cells.

At five weeks of age, he was seen at the outpatient neonatal follow-up clinic. He was doing well, and gaining weight and his physical examination was unremarkable. He was reviewed by a pediatric cardiologist; his ECG and echocardiography were normal. At four months of age, he presented at a routine planned follow-up. He had a history of fever and cough two days before his visit. Physical examination revealed severe pallor, lethargy, and hepatosplenomegaly. Urgent full blood count showed bi-cytopenia (anemia and thrombocytopenia) and leukocytosis; Hb 4.1 (RR 10.5–13.5 g/dL), WBC 25 × 10^9^ [RR 6.0–17.5 × 10^9^/L], neutrophils 12.5 x10^9^/[RR 6.0–17.5 x10^9^/L, platelets 52 × 10^9^ [RR 150–450 × 10^9^/L], and reticulocytosis of 16.1 % [RR 0.2–2.0 %]. The infant was admitted for further investigations and management. Blood film showed anemia and thrombocytopenia (large platelet forms were noted) with significant schizocytosis and polychromasia. A leukoerythroblastic picture with a left shift up to the blast stage (1 % of WBC) was noted. Reactive lymphocytes were seen (Figure 1). Markers of hemolysis showed elevated LDH 3580 [RR: 120–300 U/L], elevated total bilirubin 41 [RR: 0–17 μmol/L], low haptoglobin <0.1 [RR: 0.3–2.0 μmol/L], and DAT negative. The G6PD deficiency screening was negative, hemoglobin variant analysis was normal, and hereditary spherocytosis screening by flow cytometry was normal. Investigations for possible infectious etiology were all negative, including Hepatitis (B, C, E), CMV, EBV, HSV, VZV, and parvovirus.

Other investigations showed a normal coagulation profile, renal function tests and electrolytes, and elevated ferritin 762 ng/mL [RR: 12–327]. Workup to evaluate for congenital vs. acquired TTP revealed low ADMTS 12 activity 0.2 % [RR: 60.6–130.6] and negative anti-ADAMTS 13 antibodies. Congenital TTP was finally confirmed by the identification of a homozygous variant c.1192C>G p.(Arg398Gly) in the *ADAMTS 13* gene.

The infant was transfused with PRBCs considering the severe anemia. After sending ADAMTS 13 level and antibodies, the infant received daily FFP for four days. The anemia responded well to PRBC transfusion. The platelet count gradually improved with the FFP transfusions. On discharge after four days, the full blood count showed Hb 9.5 g/dL, and Platelets 182 × 10^9^/L. The infant was followed up every  3 weeks to assess the need for FFP, he required FFP transfusion almost every 3 weeks to maintain stable counts. His developmental milestones were regularly monitored, which so far has been appropriate for his age.

## Discussion

The most common clinical presentations for congenital TTP in neonates are neonatal jaundice and thrombocytopenia [9], 10]. Jaundice is due to microangiopathic hemolytic anemia, often requiring an exchange transfusion. Individuals who survive the neonatal period later present with recurring episodes of thrombocytopenia and hemolytic anemia, which may be associated with variable degrees of acute kidney injury [10]. The episodes can occur spontaneously but are often triggered by physiological stressors and infections [10]. Pulmonary involvements in TTP have been rarely described [11]. In our case, the patient presented initially with severe pathological neonatal indirect hyperbilirubinemia managed with intensive phototherapy and was planned initially for exchange transfusion. However, within a short time, he developed hypoxemic respiratory failure, hemolytic anemia and DIC. He was thought to have severe clinical sepsis and septic shock with secondary pulmonary hypertension, DIC and AKI. After surviving his severe neonatal course, he presented after a few months with a clinical picture of post-infectious microangiopathic hemolytic anemia, and thrombocytopenia. Due to the significant schizocytosis in the blood film, c.TTP was suspected. The diagnosis was confirmed with a very low ADAMTS 13 level in the absence of the ADAMTS 13 inhibitors and the presence of a homozygous variant c.1192C>G p.(Arg398Gly) in the *ADAMTS 13* gene.

Such dramatic neonatal presentation of c.TTP has been previously described and has been associated with a high risk of mortality. Sharma D. et al. described a neonate who presented at 40 h with jaundice, red urine, respiratory distress, and failure. The infant deteriorated and died of respiratory failure and refractory shock. The ADAMTS13 deficiency and gene mutation were confirmed later [12]. Similarly, Yang L. et al. reported three siblings with c.TTP who presented after birth with severe jaundice, anemia- and thrombocytopenia, all deteriorated rapidly and died. Whole exome testing confirmed a compound heterozygous variant in the *ADAMTS13* gene [13].

Wang and Zhao reported a neonate who presented within 1 h after birth with respiratory distress and profound thrombocytopenia and died with systemic hemorrhage. Genetic testing showed that the neonate carried a compound heterozygous mutation in the *ADAMTS13* gene. The infant had two previous siblings who died of similar clinical presentation [14].

Pulmonary involvement with PPHN causing hypoxic respiratory failure complicated the initial presentation of our patient. PPHN has also been described by Tsuji N. et al., who reported a neonate with c.TTP presented soon after birth with severe hemolysis and PPHN, which improved with exchange transfusion. However, the genetic testing identified a compound heterozygous mutation in the *ADAMTS13* gene as compared to the homozygous variant seen in our patient [15]. PPHN is postulated to be related to the nitric oxide-scavenging effect of free hemoglobin and formulation of platelet thrombi in the pulmonary vasculature [16].

The underlying ADAMTS13 deficiency in c.TTP can be addressed by treatment with FFP, which contains physiological amounts of the ADAMTS13 enzyme [17]. Thus, prophylactic administration of FFP can be an effective management strategy for this condition. To this end, Barbot et al. describe a case of a 14-year-old girl with intractable TTP that initially improved with FFP treatment but consistently relapsed. She was, therefore, given prophylactic FFP infusions at a dose of 10 mL/kg once every three weeks, which prevented further TTP relapse during the 10 years of follow-up reported in her case [18]. In our report, we believe that the FFP transfusions given as part of supportive care to treat DIC contributed greatly to surviving his initial acute neonatal presentation. After establishing the diagnosis of c.TTP, he was kept on regular FFP every three weeks. It was noticed he maintained without major episodes of TTP. However, it has been observed he required FFP more frequently during the infection periods, needing FFP every two weeks instead of three weeks.

In conclusion, we described an infant with congenital TTP, presented with very early neonatal indirect hyperbilirubinemia, thrombocytopenia, and hemolytic anemia, complicated with hypoxemic respiratory failure due to PPHN, acute kidney injury, and DIC. He was initially thought to have as complicated early neonatal sepsis and survived with supportive care including FFP and iNO. The diagnosis of congenital TTP was suspected after his later presentation with infectious-stress-induced acute hemolytic anemia and thrombocytopenia. This case highlights the importance of keeping the diagnosis of congenital TTP as one of the possibilities in neonates with indirect hyperbilirubinemia and thrombocytopenia and evidence of intravascular hemolysis.
